# Epitome, in English, of Hippocrates and Galen

**Published:** 1845-11

**Authors:** 


### HIPPOCRATES AND GALEN REDIVIVUS.

Our old master, the medical antiquary of America, Dr. John Redman Coxe, of Philadelphia, has announced an epitome, in our own language, of the works of the Father of Medicine, and his Roman Commentator, Galen. We hope our learned and venerable friend may meet with encourgement in his enterprise, and to aid in its furtherance, we give the following paragraphs from his circular: D.

"Although the works of Hippocrates and of Galen have, through a series of years, passed through numerous editions in Europe, both in Greek and Latin, as also in the French, yet, with the exception of a very few of the tracts or treatises of the former, none of their writings have appeared in the English language. Spoken of every where as the Fathers of the Medical Profession, and familiarly adverted to, even ex-cathedra, as though they had been seduously perused, and the contents of their extensive folios fully investigated and familiarly known; it is nevertheless the fact, that few of the profession in the Western Hemisphere have ever seen them, and fewer still have considered it worth their while to consult those pages of a distant era, in the nearly obsolete idioms in which they are almost solely to be found.

"Such being the case, I have been led to the belief, after many years' acquaintance with their singular worth and interesting character, that an abstract or epitome of the writings of those illustrious men might prove acceptable to the physicians of the United States, if the opportunity were offered of perusing it in the English language. Such an epitome I have prepared, and propose to put it to press if 500 subscribers of the ten thousand medical men of the Union can be obtained. It is almost impossible to state precisely the extent of the work, derived as it is from seven or eight folios; but it is believed that it can be so condensed as to be embraced in THREE, perhaps TWO, octavo volumes, according to the type, of from 500 to 600 pages each, at a price not exceeding $3 a volume.

"I need not say that it has been a work of considerable labor, yet assuredly one of infinite interest and gratification to myself; and it is chiefly from such considerations that I am induced to hope, that if printed, it will afford an equal gratification to my medical cotemporaries, and present to them, although epitomized, an adequate idea of those venerable writings, which have reached us after a lapse of more than 2,000 years.

"Physicians at a distance wishing to subscribe for it, can do so, by forwarding their names [post paid,] to Lindsay & Blakiston, Philadelphia."

